# Supplementary material for: Assessment of the Quality of post-abortion care records in 20 Public Health Facilities in Uganda: What are the gaps and how can we improve quality?
Source: PLoS One. 2025 Sep 17;20(9):e0329842. doi: 10.1371/journal.pone.0329842 (PMC12443310; doi:10.1371/journal.pone.0329842)
Supplement: Attachment — Submitted filename: PONE-D-24-54815_feedback from SA 27 Jan 2025.pdf [file pone.0329842.s002.pdf]

PLOS ONE

Review of the quality of post-abortion care records across 80 Public Health Facilities in Uganda: What are the gaps and how do we improve the data quality?

--Manuscript Draft--

**Manuscript Number:**	PONE-D-24-54815
**Article Type:**	Research Article
**Full Title:**	Review of the quality of post-abortion care records across 80 Public Health Facilities in Uganda: What are the gaps and how do we improve the data quality?
**Short Title:**	Quality of post abortion care records in public health facilities, Uganda
**Corresponding Author:**	Lynn Muhimbuura Atuyambe, MPH, PhD<br>Makerere University Faculty of Medicine: Makerere University College of Health Sciences<br>Kamplala, UGANDA
**Keywords:**	Data Quality Assessment, Review of Post-Abortion Care, Maternal Health, Reproductive Health, Uganda
**Abstract:**	Background: Effective strategies for reducing maternal mortality depend on the accuracy of data. In sub-Saharan Africa, however, weak health systems often result in significant gaps in data collection and analysis. These gaps can lead to misreporting, missing data, or double counting at health facilities, ultimately skewing the aggregated data reported by Ministries of Health. This is a big problem given that the data shapes planning and policy of the country. In this study, we assessed the data quality of Post-abortion Care (PAC) records across 80 health facilities in Uganda.<br>Methods: We retrospectively extracted data from gynecological registers in public health facilities offering PAC and selected 80 out of 7,631 facilities across Uganda using stratified random sampling from HC II to national referral hospitals. The selection was based on PAC caseload, categorized as high (over 1000 cases) or low (under 500 cases) per year, to capture distinct performance variations. High-caseload facilities face resource constraints and data overload, while low-caseload facilities struggle with underreporting and staff inexperience. Moderate caseloads were excluded to focus on extreme contexts and gain clearer insights into data quality issues. The study, conducted from June 2021 to September 2022, assessed data completeness, timeliness, and consistency using WHO guidelines.<br>Results: Health facilities achieved a 100% timeliness rate for report submissions, with all documents provided by the 7th day of each month. There was strong internal consistency amongst data elements for similar indicators. For example, we observed a positive relationship between records of women who received post-abortion care (PAC) and those who received PAFP (r =0.083, p<0.001) consistent with national health policies. However, comparing facility records with DHIS2 data revealed that outpatient records were more accurate than inpatient records, with PAC data being more inaccurate than other maternal health indicators. Further analysis by facility type indicated a higher number of case-load outliers at Health Centre IIIs and IIs (1049) compared to hospitals and Health Centre IVs, highlighting disparities in data quality across different levels of healthcare facilities.<br>Conclusion:<br>Despite high submission rates, PAC data accuracy was notably low, highlighting the need for better data management and record-keeping, particularly in lower-level health facilities. Addressing these disparities is crucial for improving maternal health outcomes, emphasizing the necessity for targeted interventions to enhance data accuracy and reliability within Uganda's health system
**Order of Authors:**	Lynn Muhimbuura Atuyambe, MPH, Ph.D.
	Justine N Bukenya, MB.Ch.B, MPH, PhD
	Samuel Etajak
	Jesca Nsungwa Sabiiti, MB.Ch.B, MMED Paed, PhD
	Richard Mugahi, MB.Ch.BMPH

Powered by Editorial Manager® and ProduXion Manager® from Aries Systems Corporation

	Paul Mbaka
	Onikepe Owolabi
	Sharon Kim-Gibbons
	Kristy Friesen
	Arthur Bagonza, MPH, PhD
**Additional Information:**	
**Question**	**Response**
**Financial Disclosure**<br><br>Enter a financial disclosure statement that describes the sources of funding for the work included in this submission. Review the submission guidelines for detailed requirements. View published research articles from *PLOS ONE* for specific examples.<br><br>This statement is required for submission and **will appear in the published article** if the submission is accepted. Please make sure it is accurate.<br><br>**Funded studies**<br>Enter a statement with the following details:<br>• Initials of the authors who received each award<br>• Grant numbers awarded to each author<br>• The full name of each funder<br>• URL of each funder website<br>• Did the sponsors or funders play any role in the study design, data collection and analysis, decision to publish, or preparation of the manuscript?<br><br>Did you receive funding for this work?	Yes
Please add funding details.<br>as follow-up to "**Financial Disclosure**<br><br>Enter a financial disclosure statement that describes the sources of funding for the work included in this submission. Review the submission guidelines for detailed requirements. View published research articles from *PLOS ONE* for specific examples.	Finding for this work was secured from Vital Strategies Inc. New York USA. How ever the study ended a year a go.

This statement is required for submission and **will appear in the published article** if the submission is accepted. Please make sure it is accurate.<br><br>**Funded studies**<br>Enter a statement with the following details:<br>• Initials of the authors who received each award<br>• Grant numbers awarded to each author<br>• The full name of each funder<br>• URL of each funder website<br>• Did the sponsors or funders play any role in the study design, data collection and analysis, decision to publish, or preparation of the manuscript?<br><br>Did you receive funding for this work?"	
Please select the country of your main research funder (please select carefully as in some cases this is used in fee calculation).<br>as follow-up to "**Financial Disclosure**<br><br>Enter a financial disclosure statement that describes the sources of funding for the work included in this submission. Review the submission guidelines for detailed requirements. View published research articles from *PLOS ONE* for specific examples.<br><br>This statement is required for submission and **will appear in the published article** if the submission is accepted. Please make sure it is accurate.	UNITED STATES - US

**Funded studies**<br>Enter a statement with the following details:<br>• Initials of the authors who received each award<br>• Grant numbers awarded to each author<br>• The full name of each funder<br>• URL of each funder website<br>• Did the sponsors or funders play any role in the study design, data collection and analysis, decision to publish, or preparation of the manuscript?<br><br>Did you receive funding for this work?"	
**Competing Interests**<br><br>Use the instructions below to enter a competing interest statement for this submission. On behalf of all authors, disclose any competing interests that could be perceived to bias this work—acknowledging all financial support and any other relevant financial or non-financial competing interests.<br><br>This statement is required for submission and **will appear in the published article** if the submission is accepted. Please make sure it is accurate and that any funding sources listed in your Funding Information later in the submission form are also declared in your Financial Disclosure statement.<br><br>View published research articles from *PLOS ONE* for specific examples.	The authors have declared that no competing interests exist.

**NO authors have competing interests**<br><br>Enter: *The authors have declared that no competing interests exist.*<br><br>**Authors with competing interests**<br><br>Enter competing interest details beginning with this statement:<br><br>*I have read the journal's policy and the authors of this manuscript have the following competing interests: [insert competing interests here]*<br><br>* typeset	
**Ethics Statement**<br><br>Enter an ethics statement for this submission. This statement is required if the study involved:<br><br>• Human participants<br>• Human specimens or tissue<br>• Vertebrate animals or cephalopods<br>• Vertebrate embryos or tissues<br>• Field research<br><br>Write "N/A" if the submission does not require an ethics statement.<br><br>General guidance is provided below. Consult the submission guidelines for detailed instructions. **Make sure that all information entered here is included in the Methods section of the manuscript.**	Ethical clearance was obtained from the Makerere University School of Public Health Research and Ethics Committee (SPHREC) reference number SPH-2022-226 as well as from the Uganda National Council of Science and Technology (UNCST) (HS2260ES). Written informed consent was sought from participants involved in the study. We also obtained written authorization to conduct the study from the Uganda Ministry of Health (MoH), district health officers, and hospital review boards provided administrative clearance.

**Format for specific study types**<br><br>**Human Subject Research (involving human participants and/or tissue)**<br>• Give the name of the institutional review board or ethics committee that approved the study<br>• Include the approval number and/or a statement indicating approval of this research<br>• Indicate the form of consent obtained (written/oral) or the reason that consent was not obtained (e.g. the data were analyzed anonymously)<br><br>**Animal Research (involving vertebrate animals, embryos or tissues)**<br>• Provide the name of the Institutional Animal Care and Use Committee (IACUC) or other relevant ethics board that reviewed the study protocol, and indicate whether they approved this research or granted a formal waiver of ethical approval<br>• Include an approval number if one was obtained<br>• If the study involved *non-human primates*, add *additional details* about animal welfare and steps taken to ameliorate suffering<br>• If anesthesia, euthanasia, or any kind of animal sacrifice is part of the study, include briefly which substances and/or methods were applied<br><br>**Field Research**<br><br>Include the following details if this study involves the collection of plant, animal, or other materials from a natural setting:<br>• Field permit number<br>• Name of the institution or relevant body that granted permission	
**Data Availability**<br><br>Authors are required to make all data underlying the findings described fully available, without restriction, and from the time of publication. PLOS allows rare exceptions to address legal and ethical concerns. See the PLOS Data Policy and FAQ for detailed information.	Yes - all data are fully available without restriction

A Data Availability Statement describing where the data can be found is required at submission. Your answers to this question constitute the Data Availability Statement and **will be published in the article**, if accepted.<br><br>**Important:** Stating 'data available on request from the author' is not sufficient. If your data are only available upon request, select 'No' for the first question and explain your exceptional situation in the text box.<br><br>Do the authors confirm that all data underlying the findings described in their manuscript are fully available without restriction?	
**Describe where the data may be found in full sentences. If you are copying our sample text, replace any instances of XXX with the appropriate details.**<br><br>• If the data are **held or will be held in a public repository**, include URLs, accession numbers or DOIs. If this information will only be available after acceptance, indicate this by ticking the box below. For example: *All XXX files are available from the XXX database (accession number(s) XXX, XXX.).*<br>• If the data are all contained **within the manuscript and/or Supporting Information files**, enter the following: *All relevant data are within the manuscript and its Supporting Information files.*<br>• If neither of these applies but you are able to provide **details of access elsewhere**, with or without limitations, please do so. For example:<br><br>*Data cannot be shared publicly because of [XXX]. Data are available from the XXX Institutional Data Access / Ethics Committee (contact via XXX) for researchers who meet the criteria for access to confidential data.*<br><br>*The data underlying the results presented in the study are available from (include the name of the third party*	Data will be available with the Makerere University Research and Ethics Committee administrator. https://sph.mak.ac.ug/research-innovations/research-and-ethics-committee

and contact information or URL).<br>• This text is appropriate if the data are owned by a third party and authors do not have permission to share the data.<br><br>* typeset	
Additional data availability information:	Tick here if the URLs/accession numbers/DOIs will be available only after acceptance of the manuscript for publication so that we can ensure their inclusion before publication.

Powered by Editorial Manager® and ProduXion Manager® from Aries Systems Corporation

Manuscript

Click here to access/download;Manuscript;Manuscript_DQA_MakSPH_03_12

Title:

Review of the quality of post-abortion care records across 80 Public Health Facilities in Uganda: What are the gaps and how do we improve the data quality?

Short title:

Review of post-abortion care data quality in 80 health facilities across Uganda.

Lynn M. Atuyambe[1]*, Justine Bukenya[1], Samuel Etajak[2], Jesca Nsungwa Sabiiti[3], Richard Mugahi[3], Paul Mbaka[3], Onikepe Owolabi[4], Sharon Kim-Gibbons[4], Kristy Friesen[4], Arthur Bagonza[1]

[1]Department of Community Health and Behavioral Sciences, Makerere University College of Health Sciences, School of Public Health, Kampala, Uganda

[2]Department of Disease Control and Environmental Health, Makerere University College of Health Sciences, School of Public Health, Kampala, Uganda

[3] Ministry of Health, Uganda

[4] Vital Strategies, USA

This study was supported by an anonymous donor.
*Corresponding author
Email: atuyambe@musph.ac.ug

ABSTRACT

Background: Effective strategies for reducing maternal mortality depend on accuracy of data. In sub-Saharan Africa, however, weak health systems often result in significant gaps in data collection and analysis. These gaps can lead to misreporting, missing data, or double counting at health facilities, ultimately skewing the aggregated data reported by Ministries of Health. This is a big problem given that the data shapes planning and policy of the country. In this study, we assessed the data quality of Post-abortion Care (PAC) records across 80 health facilities in Uganda.

Methods: We retrospectively extracted data from gynecological registers in public health facilities offering PAC and selected 80 out of 7,631 facilities across Uganda using stratified random sampling from HC II to national referral hospitals. Selection was based on PAC caseload, categorized as high (over 1000 cases) or low (under 500 cases) per year, to capture distinct performance variations. High-caseload facilities face resource constraints and data overload, while low-caseload facilities struggle with underreporting and staff inexperience. Moderate caseloads were excluded to focus on extreme contexts and gain clearer insights into data quality issues. The study, conducted from June 2021 to September 2022, assessed data completeness, timeliness, and consistency using WHO guidelines.

Results: Health facilities achieved a 100% timeliness rate for report submissions, with all documents provided by the 7th day of each month. There was strong internal consistency amongst data elements for similar indicators. For example, we observed a positive relationship between records of women who received post-abortion care (PAC) and those who received PAFP ($r = 0.083$, $p < 0.001$) consistent with national health policies. However, comparing facility records with DHIS2 data revealed that outpatient records were more accurate than inpatient records, with PAC data being more inaccurate than other maternal health indicators. Further analysis by facility type indicated a higher number of case-load outliers at Health Centre IIIs and IIs (1049) compared to hospitals and Health Centre IVs , highlighting disparities in data quality across different levels of healthcare facilities.

Conclusion:

Despite high submission rates, PAC data accuracy was notably low, highlighting the need for better data management and record-keeping, particularly in lower-level health facilities. Addressing these disparities is crucial for improving maternal health outcomes, emphasizing the necessity for targeted interventions to enhance data accuracy and reliability within Uganda's health system.

Keywords: Data Quality Assessment, Review of Post-Abortion Care, Maternal Health, Reproductive Health, Uganda

Introduction

Global targets set under SDG3 aim at reducing the global maternal mortality ratio (MMR) to below 70 per 100,000 live births by 2030 [1]. It is widely acknowledged that routine health information systems should capture specific maternal indicators to monitor progress towards these global targets. In Uganda, the Ministry of Health and its partners advocate for comprehensive understanding and continuous monitoring of factors contributing to maternal morbidity and mortality by collecting high-quality data from healthcare facilities transmitted via the DHIS2 system. The current maternal mortality rate stands at 189 per 100,000 live births [2], with abortion contributing significantly to this figure. Recent data reveals that severe abortion morbidity ratio is 2,063 per 100,000 live births, with a near-miss ratio of 938 and a mortality ratio of 23 per 100,000 live births. The Eastern region faces the most severe outcomes, largely due to insufficient readiness of healthcare facilities to provide adequate postabortion care [3].

Despite several interventions, limited data quality assessments have been conducted in areas such as maternal and reproductive health, often overlooking post-abortion care data entirely. Notably, restrictive abortion laws in Uganda pose a significant challenge to collecting reliable PAC data [4, 5]. Good quality post-abortion care (PAC) data plays a crucial role in tracking the prevalence and severity of admissions for abortion-related diagnoses within communities [6, 7] and thus understanding and proposing strategies to prevent abortion-related maternal morbidity and mortality[8]. However, in low and middle-income countries (LMIC) like Uganda, where routine health data from the health system is sparse and often of questionable quality, collecting high-quality PAC data remains highly challenging due to misinterpreted restrictive anti-abortion laws.

Although DHIS2 has been implemented at the district level to enhance data reporting and management, many lower-level health facilities rely on traditional paper-based systems. This dependence creates several challenges: paper-based methods are susceptible to errors, such as incomplete records and inaccuracies. As a result, reports from these lower-level facilities often contain mistakes, compromising the data quality ultimately reported to district and national levels. This shift from paper to digital systems has been slowed by many health workers lacking computer literacy and limited training opportunities due to resource constraints. Moreover, the scarcity of computers and poor internet access further complicate the situation, making it difficult for these health facilities to transition to digital systems effectively [9-11].

By examining the quality of post-abortion care data within a constrained policy environment, this research aims at informing planning and budgeting for PAC services which are resource-intensive compared to other maternal interventions that have received considerable national attention like family planning. The primary objective of this study was to evaluate the quality of routinely collected data documented in DHIS2 across 80 randomly selected health facilities in Uganda. Utilizing the World Health Organization's data quality review toolkit [12]. The assessment focused on dimensions such as completeness, timeliness, and consistency. This will eventually facilitate planning and decision-making based on improved data. The findings from this assessment provide insights into the status of PAC data across public health facilities, and will provide important evidence for policymakers to make decisions that address maternal morbidity and complications related to abortion. It also provides insight into aspects of how post-abortion care is managed in communities where comprehensive abortion care is restricted.

Methods

Study setting

Uganda has a population of 45.9 million people with the majority being female [13]. According to the 2022 Demographic and Health Survey estimates, Uganda's MMR stood at 189/100,000 live births, showing a 44% decline from 336 in 2016

Uganda has a total of 8,639 health facilities, 7,631 of which are predominantly public and provide Post-Abortion Care (PAC). The public health facilities are organized hierarchically. At the base are community health workers (CHWs) or volunteers. Above them are Health Centre IIs (HC IIs), which offer preventive, promotive, and outpatient curative health services, outreach care, and emergency services to a population of 5,000 people per center.

Next in the hierarchy are HC IIIs which provide preventive, promotive, outpatient curative, maternity, inpatient health services, and laboratory services for a catchment population of 20,000 people. Following HC IIIs are HC IVs serving 100,000 people with a broader range of services including emergency surgery, blood transfusion, and comprehensive laboratory services. District hospitals come next, covering a catchment of 500,000 people. They offer all the services HC IVs provide, along with in-service training, consultation, and research for the communities. Regional Referral Hospitals serve a population of 2,000,000 and provide specialized services such as psychiatry, ENT, ophthalmology, dentistry, intensive care, radiology, and pathology, in addition to the services offered by district hospitals.

At the top of the hierarchy are the National Referral Hospitals, which cater to a population of 10,000,000 people. These hospitals offer comprehensive specialist services and are heavily involved in teaching and research [14].

In Uganda, PAC is only provided by a gynaecologist or medical doctor who are mostly found at Health Centre IVs, district hospitals, regional referrals and National referrals. In some instances, however, doctors are found at Health Centre IIIs. With the advent of manual vacuum aspirators, the World Health Organization (WHO) recommends training of cadres such as Auxiliary nurses, nurses, midwives, and clinicians that can be found at lower-level health facilities to provide PAC. [15]

Sample size calculation and sampling

We determined the sample size for facilities included in this assessment by obtaining the total number of health facilities that offer post-abortion care in Uganda. In total, 7,631 public health facilities out of the 8,639 health facilities were engaged in PAC. We then calculated the probability (p) of finding a health facility that offers PAC. This was calculated as (7,631/8,639*100) giving us a p=0.88 using the Kish Leslie formula. We used the calculated prevalence (p), a margin of error of 1.96 at a 95% confidence level. We determined 162 health facilities as the total sample size of health facilities to be visited for the DQA.
However, since the number of health facilities offering PAC services is finite (the number is limited and known), the sample size was adjusted using the sample size calculation for finite populations. Ultimately, we ended up with 79 health facilities. Due to proximity, we merged the Southern and Central regions, giving us four regions. With the four regions, we rounded off to 80 facilities, with 20 health facilities per region for better comparison.
From each of the four regions (Northern, Western, Eastern, and Central), we selected districts based on two criteria. The first criterion was districts with a high number of PAC cases (where high referred to more than 1000 cases per year). The second criterion was districts with low PAC cases (where low refers to less than 500 cases per year) as per the DHIS2 from January 2021 to

December 2021. Districts with caseloads in between the high and low caseloads were not part of the study because the investigators wanted districts with clear-cut differences as far as caseloads were concerned. Figure 1 shows the map of Uganda with the number of cases per year used to guide the random sampling of districts. This was followed by a random selection of two districts per region meeting the above criteria. Districts meeting the criteria above had all health facilities offering PAC arranged in alphabetical order. Sampling proportionate to the health facility numbers then ensued. Districts selected as having high caseloads included: Gulu, Mbarara, Mityana, and Kamuli, while districts with low caseloads included: Nwoya, Kiruhura, Nakasongola, and Pallisa.

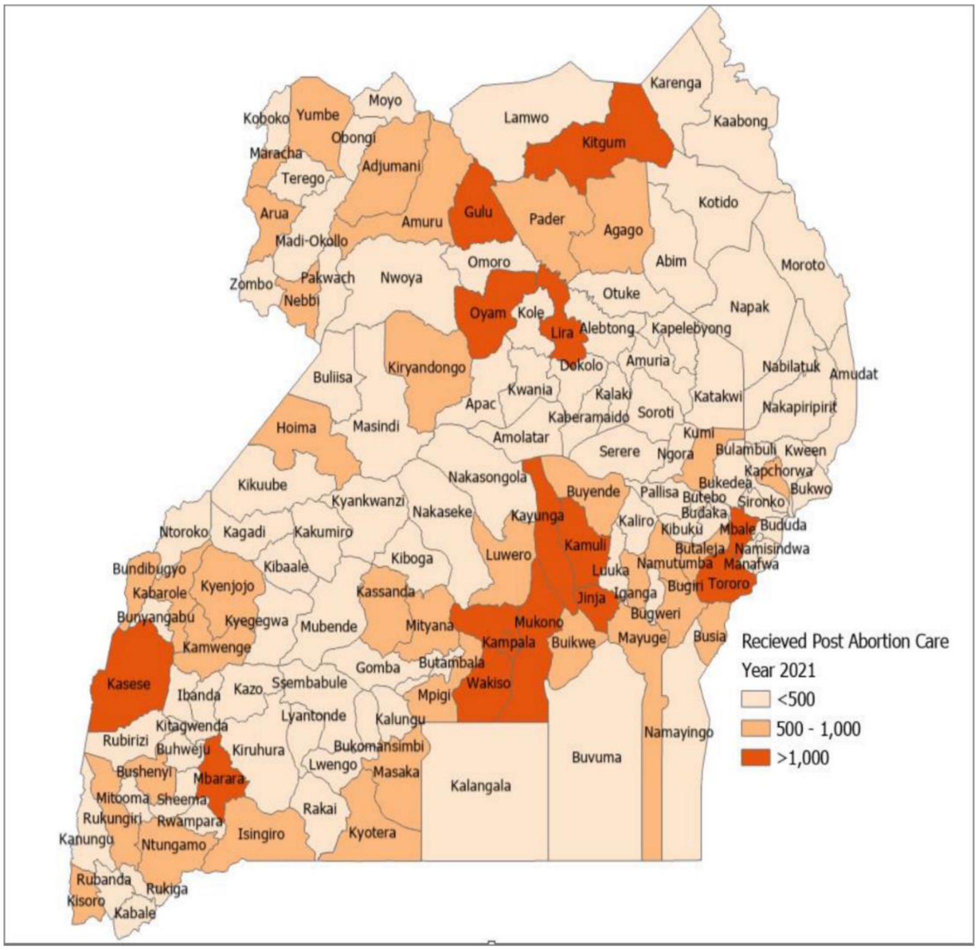

Figure 1: Map of Uganda showing the number of PAC cases per district for the year 2021

Data sources

For this assessment, data were obtained from two primary sources: facility-reported records within the District Health Information System 2 (DHIS2) and an independent facility survey. In the DHIS2, the only indicators available for Post-Abortion Care (PAC) were related to abortions due to Gender-Based Violence (GBV), abortions from other causes, and the number of post-abortion women who received family planning. However, we also reviewed additional indicators such as antenatal care (ANC) visits, total maternity ward admissions, total births, live births, and live births at discharge, as the same cadre of healthcare workers manages these. Our aim was to understand why there are discrepancies in the accuracy of some indicators, despite being documented by the same staff in health facilities.

A trained research team extracted data from the facilities' paper-based outpatient, maternity and delivery registers.

Our data collection started on October 1st 2022, and concurrently, investigators retrieved data from DHIS2. This data abstraction process, encompassing both the manual registers and DHIS2, spanned two months. Data was extracted from June 2021 to September 2022, after which a comparative analysis ensued between the data documented in the paper-based registers and the information extracted from DHIS2. Variables from which the data were extracted are shown in Table 1.

Table 1: Maternal and Newborn indicators used for comparison with PAC Data

Maternal and Newborn Health data elements	Uganda's routine health information system	
	Facility register	DHIS2
Post Abortion Family Planning	X	X
**Abortions**		
Gender-Based Violence (GBV)	X	X
Other causes	X	X
Incomplete evacuations	X	X
ANC 1st visit for women	X	X
ANC 4th visit for women	X	X
ANC 4+ visits for women	X	X
Total ANC contacts/visits (new clients + re-attendances)	X	X
**Admissions**		
Deliveries in unit - Total	X	X
Deliveries in unit - Live births	X	X
Deliveries in unit - Fresh stillbirth - Total	X	X
Deliveries in unit - Macerated stillbirth - Total	X	X
Malaria in pregnancy (cases)	X	X
High blood pressure in pregnancy (cases)	X	X

Data quality assessment

The quality of DHIS2 data was evaluated based on three routine data quality dimensions defined by the World Health Organisation's data quality review toolkit: completeness and timeliness, internal consistency, and external consistency [12]. Table 2 details the evaluation criteria for each quality metric and the data sources utilized. In addition, a stratified analysis was performed according to the types of healthcare facilities.

Table 2: PAC data quality metrics used and their definitions

**Post-abortion care (PAC) data quality metrics and data sources reviewed**			
**PAC data quality metric**	**Definition of the indicator and the WHO recommendation**	**Source(s)**	**Facilities**
**Data quality dimension 1: Completeness and timeliness of PAC data**			
Completeness of facility reporting in DHIS2<br>The extent to which health facilities in the district submitted monthly summary reports containing PAC data to the district HMIS focal person	Total number of health facilities expected to submit monthly reports with PAC data versus actual number of health facilities submitting reports with PAC data<br><br>*WHO recommends 75% or higher reporting*	DHIS2 records extracted at district level	33 Health Centre II<br>38 Health Centre IIIs<br>4 Health Centre IV and<br>5 Regional Referral Hospitals
Timeliness of facility reporting PAC data in DHIS2<br>The extent to which health facilities in the district submitted monthly summary reports containing PAC data before the specified timeline	Number and percentage of health facility reports actually submitted on time<br><br>*WHO has no specific guidance on the timeliness of facility reporting*	Records from the District HMIS focal person	33 Health Centre II<br>38 Health Centre IIIs<br>4 Health Centre IV and<br>5 Regional Referral Hospitals
Completeness of Indicator data in DHIS2<br>Extent to which select PAC indicator data within submitted reports contained a non-missing (or non-zero) value.	Number and percentage of non-missing values for a given PAC indicator in expected monthly reports<br><br>There should be no missing values for a given indicator in 90% or more monthly reports	Records from the District HMIS focal person	33 Health Centre II<br>38 Health Centre IIIs<br>4 Health Centre IV and<br>5 Regional Referral Hospitals
**Data quality dimension 2: Internal consistency of PAC data**			
Consistency over time<br>The extent to which PAC indicator data exhibits similar patterns as in previous years	Ratio of the value of the indicator for the reference year to the end of the preceding 3 years<br><br>Ratio of the value of the indicator for the reference year should be within ±33% of the mean of the preceding 3 years	DHIS2	33 Health Centre II<br>38 Health Centre IIIs<br>4 Health Centre IV and<br>5 Regional Referral Hospitals

Outliers in reference year			
Extent to which the values reported for a given PAC indicator are extreme and potentially implausible	Number of moderate outliers (±2 - 3 SD from the mean) and extreme outliers (±3 SD from the mean) of monthly values during the reference year<br><br>Value of the indicator should be within ±2 SD from the mean	DHIS2	33 Health Centre II<br>38 Health Centre IIIs<br>4 Health Centre IV and<br>5 Regional Referral Hospitals
Accuracy I: Consistency between related data			
The extent to which the values for two or more PAC indicators exhibit the predicted relationship	Ratio for values of indicator pairs that have a predictable relationship<br><br>Indicator pairs that should be roughly equal should be within ±10% of each other.	DHIS2	33 Health Centre II<br>38 Health Centre IIIs<br>4 Health Centre IV and<br>5 Regional Referral Hospitals
Accuracy II: Consistency between original facility registers and reported data in DHIS2			
The extent to which values for given PAC indicators agree between two internal data sources	Ratio of indicator values in original facility register count to facility monthly summary report in DHIS2<br><br>Indicator value in the original facility register count and facility monthly report in DHIS2 should be within ±10% of each other	DHIS2 and Facility registers	33 Health Centre II<br>38 Health Centre IIIs<br>4 Health Centre IV and<br>5 Referral Hospitals

Notes: PAC = post-abortion care, SD = standard deviation
*WHO thresholds were used as cut-offs for reporting good data quality for this survey.
**For the four-year period that was studied, downloaded health facility data from Uganda's DHIS2 did not distinguish between missing values and true zero values; both are presented as missing values.

The initial comparison of raw data to the DHIS2 data from 80 health facilities from 2018 to 2021 revealed that the data did not meet the WHO's recommended accuracy rate of 75%. The highest accuracy for outpatient department (OPD) records was 13.3% in 2020, while inpatient records peaked at 10.7% accuracy in 2019. When considering OPD and inpatient records together, the

overall accuracy was 10.5% in 2020. These low accuracy levels were linked to missing records in some health facilities, primarily due to insufficient storage practices. To validate the data from the initial assessment, which had significant gaps due to missing records at certain health facilities, a follow-up visit was conducted at 20 health facilities. Data for the last seven months of 2021 and the last six months of 2022 were collected. These months were purposively chosen because the financial year in Uganda begins in June, and the authors wanted to rule out challenges associated with financial shortfalls that often occur before the beginning of the financial year. Such challenges may include but may not be limited to; inadequate essential resources needed for data collection and management, such as paper for records, computers, or even staffing. Without these resources, facilities may struggle to maintain accurate and complete records. Budget constraints can lead to delayed salary payments, which may lead to reduced data collection and entry diligence.

Ethical approval

Ethical clearance to conduct the assessment was sought from the Makerere University School of Public Health Research and Ethics Committee (SPHREC) reference number SPH-2022-226 as well as from the Uganda National Council of Science and Technology (UNCST) (HS2260ES). Informed consent was sought from participants involved in the study before the commencement of the assessment. The Ministry of Health (MoH), district health officers, and hospital review boards provided administrative clearance to assess the selected districts, cities, and health facilities. We also obtained separate administrative clearance from the new cities (Gulu and Mbarara), formed on July 1, 2020. Informed consent was sought from all health management information personnel and health facility in-charges. For medical records, our research teams had

no access to information that could identify individual participants during or after data collection in this study.

Results

Completeness of District Reporting for PAC Data Indicators

Upon analysing the completeness of report submissions across individual districts, we found Mbarara City achieved 100% while Gulu City had a submission rate of 92%. The calculation of submission rates was based on records authenticated at both the district level and by the HMIS focal person at the district headquarters, as depicted in Figure 1.

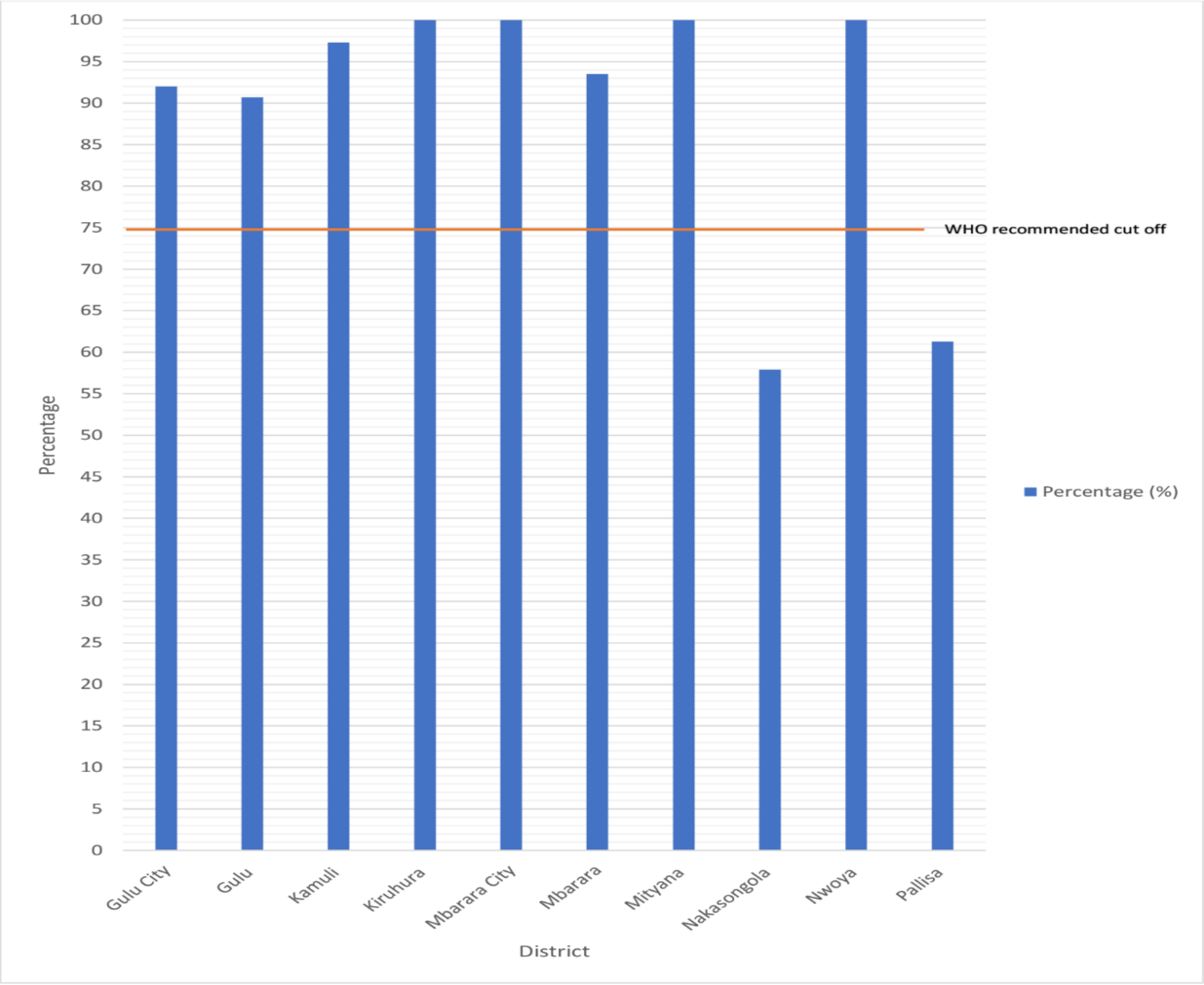

Figure 1: Average completeness reporting by district

Figure 1 shows that Pallisa (61.3%) and Nakasongola (57.9%) did not meet the World Health Organisation's (WHO) recommended 75% target for how complete their monthly reports should be.

Completeness and timeliness of facility reporting

We examined health facilities' data submissions to assess the completeness and timeliness of their reports. For completeness, we verified whether all health facilities required to send PAC data to the district's main health information officer did so and whether this data was subsequently reflected in the Ministry of Health's DHIS2 system. For timeliness, we monitored if these facilities submitted their reports by the 7th of each month and if districts forwarded this data to the Ministry of Health by the 15th of the same month.

We evaluated the data's completeness by reviewing the HMIS registers at the district level. We then used the DHIS2 database to observe whether PAC data, including the expected number of PAC cases from health facilities, were correctly sent to the district's health information person throughout the year, showing this as a rate or percentage.

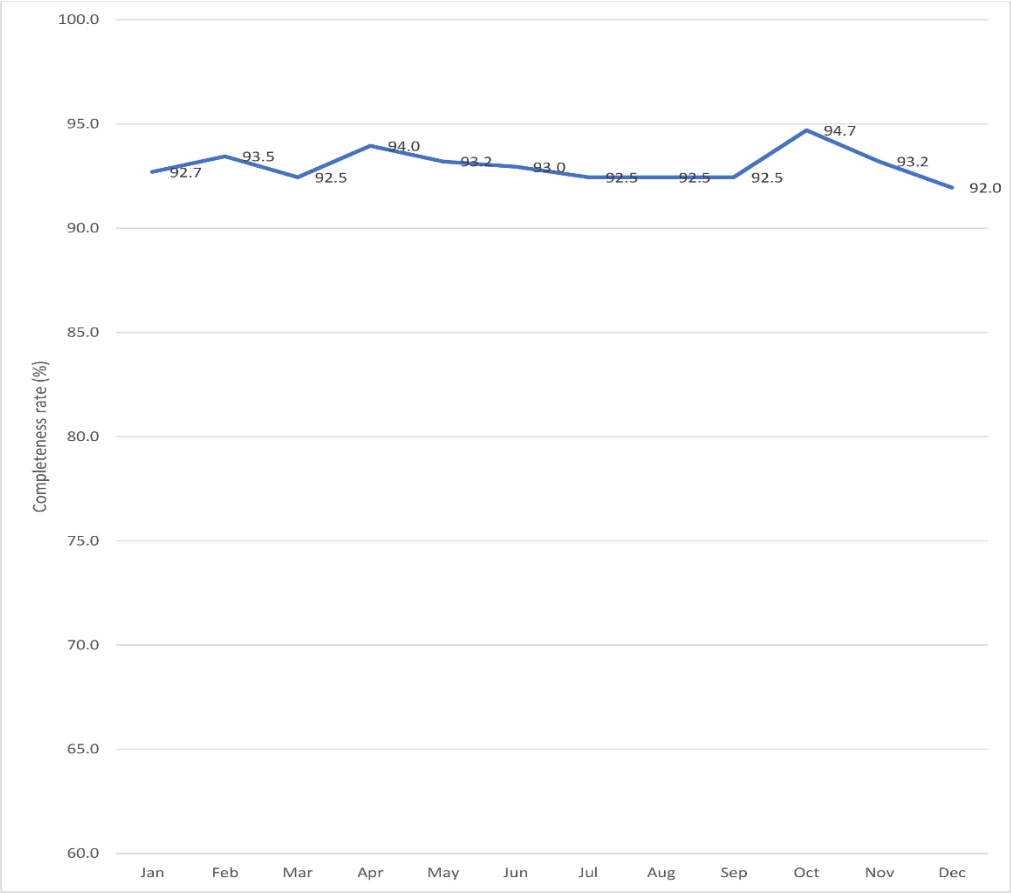

Figure 2: Overall trends in completeness of reporting by sampled health facilities

From the health facilities randomly sampled, on average, the overall completeness rate was above 90% every month, as seen in Figure 2. Additionally, when it comes to sending monthly reports with Post-Abortion Care (PAC) data, all the selected health facilities consistently submitted their reports by the 7th of each month achieving a 100% rate of timely submissions.

Consistency over time

This metric highlights the stability of crucial maternal indicators for 2021 against the preceding three years. The use of post-abortion family planning (PAFP) as the base measure for post-abortion care is informed by the Essential Maternal and Newborn Clinical Care guidelines for

Uganda which mandates that women receiving post-abortion care should also get family planning services [16].

Table 3: Consistency of reporting maternal indicators over time

	Year 1, Jan 2018-Dec 2018	Year 2, Jan 2019-Dec 2019	Year 3, Jan 2020-Dec 2020	Year 4, Jan 2021-Dec 2021	Mean of Year 1-Year 3	Consistency (Ratio of Year 4 to Mean of Year 1-Year 3)
**Main denominator**						
PAFP	0	0	205	438	-	-
**Abortions**						
GBV	16	16	37	45	23	**1.96**
Other causes	1487	1299	4097	3918	2294.33	**1.71**
Incomplete evacuations	0	0	1019	1200	-	-
ANC 1st visit for women	41421	44704	48719	73031	44948	**1.62**
ANC 4th visit for women	17271	21824	26673	33205	21923	**1.51**
ANC 4+ visits for women	5126	10237	18976	20461	11446	**1.79**
Total ANC contacts/visits (new clients + re-attendances)	117941	138457	161532	175926	139310	1.26
Admissions	33637	38231	43580	50751	38483	1.32
Deliveries in unit - Total	26466	29532	34398	40767	30132	1.35
Deliveries in unit - Live births	25976	29103	27264	36630	27448	1.33
Deliveries in unit - Fresh stillbirth - Total	242	252	281	289	258	1.12
Deliveries in unit - Macerated stillbirth - Total	288	272	283	304	281	1.08
Malaria in pregnancy (cases)	1746	3155	3257	2491	2719	0.92
High blood pressure in pregnancy (cases)	188	262	318	228	256	0.89

*According to WHO guidance, ratios <0.67 or >1.33 indicate reported data in DHIS2 for the reference year was inconsistent with the mean of the preceding 3 years.

Table 3 shows that for the first year (January 2018 to December 2018) and the second year (January 2019 to December 2019), there were no records for PAFP and incomplete evacuations.

A consistency check for other maternal indicators revealed discrepancies. The figures for abortions due to gender-based violence (1.96), abortions for other reasons (1.71), the first antenatal care ANC) visit for women (1.62), the fourth ANC visit for women (1.51), and the ratio for women attending more than four ANC visits (1.79) all exceeded the World Health Organisation's recommended threshold of 1.33 for consistent data. This suggests that the data reported in DHIS2 for the year 2021 did not match the average of the previous three years for these specific maternal health indicators.

Internal consistency of reported data by checking for outliers in DHIS2 Data

The integrity of the data sent to the DHIS2 was verified by identifying any outliers and recording their extent. Upon reviewing the data at the district level, it was observed that the PAC indicator presented the most significant number of outliers. Among all indicators evaluated, Mityana district reported the highest frequency of outliers, whereas Mbarara district had the fewest as detailed in Table 4.

Table 4: Case number of outliers by district for 5 STD from the median

District	ANC 1	ANC 4	ANC total	Admissions	Live births	PAC	Post-abortion FP	Freq.
Gulu City	20	31	26	48	47	51	7	192
Gulu District	2	9	10	57	52	146	3	288
Kamuli District	0	1	0	0	0	128	13	432
Kiruhura District	0	0	1	0	0	117	3	480
Mbarara City	0	4	0	120	118	37	5	288
Mbarara District	12	2	0	7	0	65	1	432
Mityana District	46	42	46	48	35	239	32	480
Nakasongola District	18	11	36	56	52	186	9	480
Nwoya District	18	46	38	107	89	230	12	468
Pallisa District	0	4	1	48	43	217	5	456

Further analysis was conducted based on the type of health facility. Table 5 indicates that Health Centers II and III exhibit a significantly higher number of outliers across most health indicators than Hospitals and health Center IVs. Notably, post-abortion care (PAC) has a substantial number of outliers in these lower-level facilities, suggesting potential issues with the accuracy of cadre documentation. Similarly, high outliers are observed in live births and admissions at Health Centers II and III, raising concerns about data reliability from these facilities. In contrast, hospitals demonstrated better reporting accuracy, particularly in antenatal care and maternity admissions. These discrepancies in data recording at lower-level health centers suggest that Health Centers II and III may face challenges in consistently documenting key health indicators related to maternal and reproductive care.

Table 5: Case number of outliers by health facility level

Facility Level	ANC 1	ANC 4	ANC total	Admissions	Live births	PAC	Post-abortion FP	Freq.
Hospital	0	0	0	0	0	0	24	240
HC IV	1	1	0	0	0	2	9	192
HC III	1	12	3	3	9	637	35	1,896
HC II	31	70	43	799	726	412	22	1,668

Consistency of reported data in DHIS2

Additionally, we evaluated the coherence of the reported data by investigating the relationship between women seeking post-abortion care and those obtaining post-abortion family planning services. We also inquired if there was a link between women attending their first and fourth antenatal care (ANC) visits. The findings are presented in Table 6.

Table 6: Correlation analysis predicting post-abortion family planning and PAC

Predictor Variable	Pearson Correlation	*P*-Value
Post-abortion family planning vs outpatient PAC cases	0.113	0.00
Post-abortion family planning vs inpatient PAC cases	0.064	0.004
Post-abortion family planning vs all PAC cases	0.083	0.00
ANC first visit vs ANC 4+ visits for women	0.236	0

A positive relationship was found between women who received post-abortion care (PAC) and those who received PAFP. This relationship was more pronounced for women who only sought outpatient PAC compared to those who utilized both outpatient and inpatient services.

Similarly, a strong correlation was observed between women attending their first and fourth antenatal care (ANC) visits, with this relationship stronger than any observed within the PAC data.

Accuracy between original facility registers and reported data in DHIS2

We compared raw data from health facility records and data retrieved from the DHIS2. The data from records are presented as percentages, showing how accurate they are.

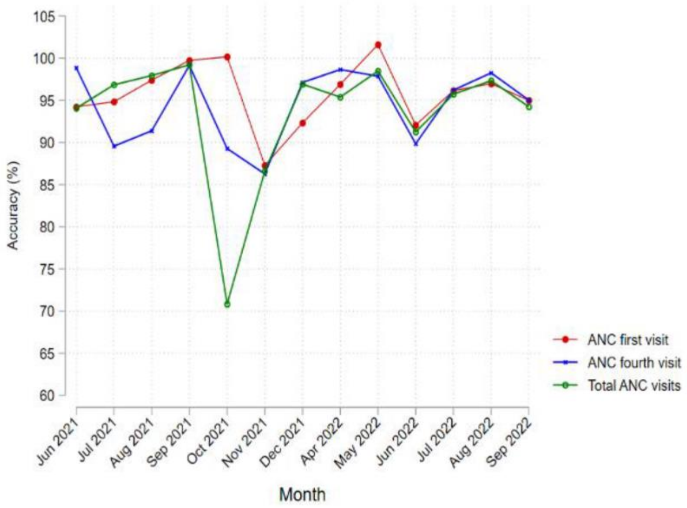

Figure 3a shows that there was general overreporting of health facility ANC data in the DHIS2, although there was some underreporting in May 2022 for the first ANC visit. The total number of ANC visits seems to be the most overreported, especially in October 2021.

This was due to high rates of data entry errors, such as double counting and incorrect aggregation of visit data, leading to inflated numbers for total ANC visits.

Figure 3a: Accuracy of reporting ANC visits June 2021-September 2022

Figure 3b shows a similar pattern of overreporting of total admissions and live birth data in the DHIS2 system. Overreporting occurred most frequently in November 2021.

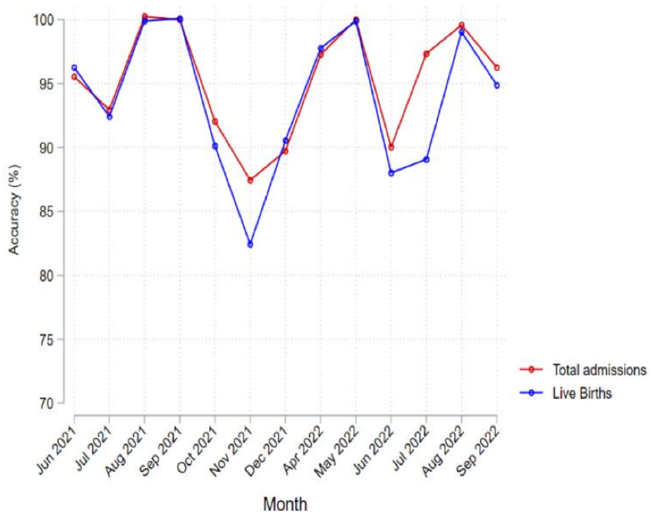

Figure 3b: Accuracy of total admissions and live births June2021-September 2022

Figure 3c shows that PAC data is the least accurate of the maternal health indicators, with over-reporting in the DHIS2.

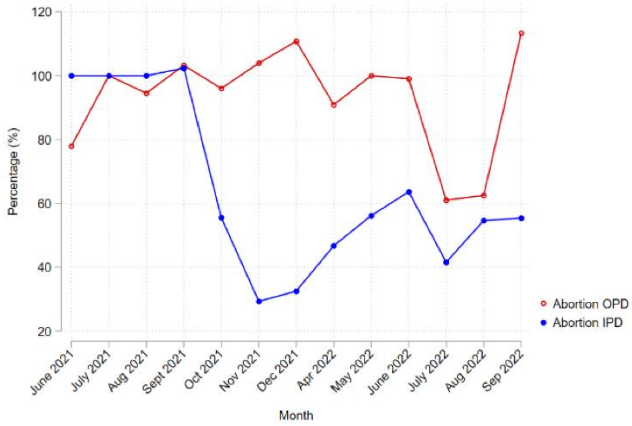

Figure 3c: Accuracy of abortion data June2021-September 2022

An in-depth analysis of record accuracy by type of health facility revealed that hospitals and Health Centre IVs maintained more precise PAC records over the years compared to Health Centre IIs and IIs, as shown in Table 7. This highlights the urgent need for training medical staff in accurate record-keeping at these lower-level facilities, which serve a substantial number of women in need of PAC.

Table 7: Accuracy of reporting rate for PAC records by health facility over time

Health Facility Type	2018	2019	2020	2021
**HC II**	8.13	6.27	12.43	20.29
**HC III**	10.35	16.21	29.67	21.13
**HC IV**	91.35	78.69	11.13	5.34
**Hospital**	38.04	81.46	49.57	55.65

Consistency between household survey and DHIS2 data

Under ideal circumstances, when the HMIS accurately captures all post-abortion care (PAC) cases presented at health facilities, the PAC values for the collected indicators should match those derived from household surveys. An exception to this rule occurs when private health providers involved in PAC service provision do not report to the HMIS system. In this assessment, household surveys were not conducted. Therefore, there was no comparison of the indicators from this assessment with those from community and household surveys.

Discussion

We evaluated the quality of PAC data reporting across various health facilities in Uganda. The average completeness rate exceeded 90% monthly, with 100% timeliness in submission. Maternal indicators like abortion due to GBV and other causes, first ANC visits, and fourth or more ANC visits were all consistently above the WHO recommended threshold of 1.33. Analysis

showed that Health Centre IIIs and IIs had more reporting inconsistencies compared to hospitals and Health Centre IVs. The study also found PAC data accuracy less reliable due to missing records and poor storage practices. These findings highlight the need for improved data accuracy and record-keeping in Uganda's health system.

Data are necessary for effective management, accountability and decision-making concerning women who have undergone abortion or face other reproductive maternal challenges. Addressing the obstacles hindering routine PAC data collection and transmission, the United Nations Sustainable Development Goal number three (SDG 3) emphasizes the necessity to enhance health system funding [17]. This would lead to increased human resources for data management and transmission within the health information system, along with improved access to healthcare providers, ultimately resulting in improved individual well-being.

Completeness and timeliness of facility reporting

The study highlights the strong performance of health facilities in reporting Post-Abortion Care (PAC) data—as part of the overall health facility data, with over 90% of the facilities reporting monthly and 100% timeliness. This reflects well on the health information systems in place. It aligns with other research emphasizing data integrity and the impact of electronic health records on healthcare delivery and policy-making [18-20]. The success suggests potential best practices for improving data quality in other areas or systems facing similar challenges, highlighting the critical role of complete and timely health data in enhancing healthcare outcomes and informing policy

Consistency of reported data

The study results also showed that Maternal indicators like abortion due to GBV and other causes, first ANC visits, and fourth or more ANC visits varied but were all consistently above the WHO recommended threshold of 1.33. These results are in tandem with a Kenyan study that evaluated the quality of maternal health data, such as ANC visits and Prevention of Mother-To-Child Transmission (PMTCT). In the study, the authors also found variability in consistency and plausibility across different health indicators, suggesting some data were more reliable than others [21]. This highlights the need for continuous data quality monitoring and evaluation across all health indicators. Health systems should implement regular data quality assessments and provide training to healthcare workers on accurate data recording and reporting practices.

Relatedly, the lower-level health facilities— HCIIs and HC IIIs experienced the highest incidence of outliers, while hospitals and HC IVs recorded the lowest number of outliers. These results mirror a Ghanaian study that assessed MCH data quality in the Cape Coast Metropolis and found that while completeness and consistency of the various maternal indicators were high, consistency varied across facilities as our study found [22]. This may necessitate lower-level health facilities getting additional resources and support to improve their data collection and reporting practices. The support may involve providing more training for staff, better data management tools, and increased supervision and oversight to ensure data accuracy.

Accuracy between original facility data and DHIS2 data

We compared raw data from health facility records and data retrieved from the DHIS2 because health facility records are the primary data source, maintained directly by healthcare providers responsible for documenting patient care and health services in real time. These records are

typically preserved as part of routine clinical and administrative processes, making them less susceptible to data loss or errors that can occur during subsequent reporting stages [23]. Moreover, health facility records are often subject to audits and verification processes, which adds an additional layer of confidence to their accuracy [24].

In contrast, data entry into systems like DHIS2 may involve multiple levels of aggregation, manual input, and potential for errors due to miscommunication, data entry mistakes, or incomplete reporting [25]. Given the structured environment in which health facility records are maintained and the direct involvement of clinical staff, we are confident that these primary records provide a more reliable and accurate reflection of the actual health services delivered, particularly in contexts where routine audits and checks are implemented.

Study results highlighted the accuracy of PAC data compared with other health indicators, emphasizing PAC data as notably less accurate, with a tendency towards over-reporting within the DHIS2 system. This finding is particularly significant as it reflects on the quality of data critical for maternal health services, which are paramount for guiding health policy and program implementation. Comparatively, the literature and reports on health data accuracy often emphasize the general challenges in achieving high fidelity in health reporting systems. Specific indicators such as those related to abortion are sometimes more prone to inaccuracies due to various factors such as the complexity of data collection processes, the sensitivity of the data in question and the level of training and resources available to health workers [26, 27]. The challenges identified in accurately capturing PAC data in Uganda echo findings from other regions and health systems that struggle with maintaining high-quality data across different health indicators [28]. The implications are that emphasis on the lower accuracy of PAC data relative to other indicators signals the need for targeted interventions to improve data collection

and reporting practices specifically for PAC services. This could involve enhanced training for healthcare providers on the importance of accurate data entry, the implementation of more user-friendly data capture tools or more rigorous data quality assurance processes.

Moreover, the observed discrepancies in data accuracy across different health facility types and among various health indicators underline the importance of a systematic approach to addressing data quality issues. For health systems, particularly with restrictive abortion laws similar to Uganda, improving the accuracy of PAC data and other health indicators is crucial for informed decision-making, resource allocation and the effective monitoring and evaluation of health interventions aimed at improving maternal health outcomes [27].

Improving the DHIS2

Currently, over 60 countries around the world use the DHIS2 platform as a one-stop Centre for collected and collated health facility data [25]. This data is used for planning and programming at the country level as a means of improving the health outcomes of populations. However, based on our study, it is difficult to say whether there are any in-built safeguards to prevent the entry of extreme values that may affect the quality of DHIS2 data. For example, the study team observed duplicate PAC entries at one referral health facility during the health facility survey. In addition, it was difficult to distinguish between a true zero and a missing value, which has also been reported in other studies [29]This makes it difficult to conduct data quality assessments using the DHIS2 in its current form for some indicators. Improving all aspects of the health system is necessary if good-quality data is to reach the DHIS2 system.

Study Strengths and Limitations

This study had a major strength in comparing paper-based monthly summaries from health facilities to their electronic DHIS2 versions. However, there was no gold standard data source for

abortions on account of stigma and so it was hard to identify the most complete source to compare DHIS2 records against, even though we were looking at PAC, which is technically not induced abortion.

During the review, some health facility records were missing due to poor storage practices. This may have impacted the comparison that was made between the health facility data and the data in the DHIS2. There is a need for the government of Uganda through the Ministry of Health to improve the storage of health facility records since these can be used as backup when unwanted people like hackers temper with the DHIS2, as it happened in the United Kingdom [31].

Finally, assessing only 80 health facilities out of the 7,631 that provide PAC in Uganda may not have provided a fully representative picture of the overall quality of post-abortion care across all health facilities in Uganda. In addition, even with a stratified sampling approach that we employed, there is a risk that the selected facilities may not have adequately captured the diversity of health facilities across different regions, levels of care, or types of communities served (urban vs rural). This could lead to biased results, especially if certain strata are underrepresented.

Conclusions

There is minimal data available on abortions and post-abortion care in the Ugandan HMIS, and the quality of data available is poor according to WHO standards. There was inconsistency of data between what was collected at health facility level with data in the DHIS2 system at the Ministry of Health. Despite high submission rates, PAC data accuracy was notably low, highlighting the need for better data management and record-keeping, particularly in lower-level health facilities. Addressing these disparities is crucial for improving maternal health outcomes,

emphasizing the necessity for targeted interventions to enhance data accuracy and reliability within Uganda's health system. We propose that districts ensure that the collected data drives the decision-making, including program design and health budgeting—this way data will be valued.

Acknowledgements

The authors thank the research assistants, data collectors, health facility in-charges, district technocrats, and the gatekeepers at the Ministry of Health Uganda.

Author Contributions

Conceptualization: Lynn Atuyambe, Justine Bukenya, Paul Mbaka, Jesca Nsungwa Sabiiti, Richard Mugahi, Sharon Kim- Gibbons and Onikepe Owolabi.

Formal analysis: Arthur Bagonza, Paul Mbaka, Lynn Atuyambe, Samuel Etajak, Justine Bukenya.

Methodology: Lynn Atuyambe, Justine Bukenya, Arthur Bagonza, Richard Mugahi, Jesca Nsungwa Sabiiti, Samuel Etajak, Onikepe Owalabi.

Resources: Sharon Kim-Gibbons, Onikepe Owolabi, Kristy Friesen

Writing – original draft: Arthur Bagonza, Lynn Atuyambe, Justine Bukenya, Samuel Etajak,

Writing – review & editing: Onikepe Owolabi, Sharon Kim-Gibbons, Kristy Friesen, Jesca Nsungwa Sabiiti, Richard Mugahi, Paul Mbaka.

References

1. WHO. THE GLOBAL HEALTH OBSERVATORY Geneva2023 [cited 2024 29/2/2024]. Available from: https://www.who.int/data/gho/data/themes/topics/indicator-groups/indicator-group-details/GHO/maternal-mortality.
2. Uganda Bureau of Statistics 2023. Uganda Demographic and Health Survey 2022. Kampala: UBOS, 2023.
3. Atuhairwe S, Gemzell-Danielsson K, Byamugisha J, Kaharuza F, Tumwesigye NM, Hanson C. Abortion-related near-miss morbidity and mortality in 43 health facilities with differences in readiness to provide abortion care in Uganda. BMJ Global Health. 2021;6(2):e003274. doi: 10.1136/bmjgh-2020-003274.
4. Kagaha A, Manderson L. Medical technologies and abortion care in Eastern Uganda. Social science & medicine (1982). 2020;247:112813. Epub 2020/02/15. doi: 10.1016/j.socscimed.2020.112813. PubMed PMID: 32058197; PubMed Central PMCID: PMCPMC7613281.

5. Kibira SPS, Stillman M, Makumbi FE, Giorgio M, Nabukeera S, Nalwoga GK, et al. Lived experiences and drivers of induced abortion among women in central Uganda. PLOS Glob Public Health. 2023;3(12):e0002236. Epub 2023/12/06. doi: 10.1371/journal.pgph.0002236. PubMed PMID: 38055682; PubMed Central PMCID: PMCPMC10699625.
6. Huber D, Curtis C, Irani L, Pappa S, Arrington L. Postabortion Care: 20 Years of Strong Evidence on Emergency Treatment, Family Planning, and Other Programming Components. Global health, science and practice. 2016;4(3):481-94. Epub 2016/08/30. doi: 10.9745/ghsp-d-16-00052. PubMed PMID: 27571343; PubMed Central PMCID: PMCPMC5042702.
7. Riley T, Madziyire MG, Owolabi O, Sully EA, Chipato T. Evaluating the quality and coverage of post-abortion care in Zimbabwe: a cross-sectional study with a census of health facilities. BMC health services research. 2020;20(1):244. doi: 10.1186/s12913-020-05110-y.
8. Arshad A, Aziz H, Shabbir G, Shakya S, Munir Z. Improving safe post-abortion care practices: A study on interventions implemented by Ipas Pakistan. Frontiers in Public Health. 2023;11. doi: 10.3389/fpubh.2023.1004381.
9. Henriksson DK, Peterson SS, Waiswa P, Fredriksson M. Decision-making in district health planning in Uganda: does use of district-specific evidence matter? Health Research Policy and Systems. 2019;17(1):57. doi: 10.1186/s12961-019-0458-6.
10. Kwesiga B, Aliti T, Nabukhonzo P, Najuko S, Byawaka P, Hsu J, et al. What has been the progress in addressing financial risk in Uganda? Analysis of catastrophe and impoverishment due to health payments. BMC health services research. 2020;20(1):741. doi: 10.1186/s12913-020-05500-2.
11. Semahegn A, Manyazewal T, Hanlon C, Getachew E, Fekadu B, Assefa E, et al. Challenges for research uptake for health policymaking and practice in low- and middle-income countries: a scoping review. Health Research Policy and Systems. 2023;21(1):131. doi: 10.1186/s12961-023-01084-5.
12. WHO. Data quality review: module 3: data verification and system assessment. Geneva: World Health Organization; 2017 2017.
13. UBOS. NATIONAL POPULATION AND HOUSIND CENSUS. Kampala: Uganda Bureau of Statistics; 2024.
14. MoH. NATIONAL HEALTH FACILITY MASTER LIST A COMPLETE LIST OF ALL HEALTH FACILITIES IN UGANDA2018.
15. WHO. Health worker roles in providing safe abortion care and post-abortion contraception. Geneva: World Health Organisation; 2015.
16. MoH. Essential Maternal and Newborn Clinical Care Guidelines for Uganda. 2022.
17. Micah AE, Su Y, Bachmeier SD, Chapin A, Cogswell IE, Crosby SW, et al. Health sector spending and spending on HIV/AIDS, tuberculosis, and malaria, and development assistance for health: progress towards Sustainable Development Goal 3. The Lancet. 2020;396(10252):693-724. doi: 10.1016/S0140-6736(20)30608-5.
18. Hoeven LRv, Bruijne MCd, Kemper PF, Koopman MMW, Rondeel JMM, Leyte A, et al. Validation of multisource electronic health record data: an application to blood transfusion data. BMC medical informatics and decision making. 2017;17(1):107. doi: 10.1186/s12911-017-0504-7.
19. Alwhaibi M, Balkhi B, Alshammari TM, AlQahtani N, Mahmoud MA, Almetwazi M, et al. Measuring the quality and completeness of medication-related information derived from hospital electronic health records database. Saudi pharmaceutical journal : SPJ : the official publication of the Saudi Pharmaceutical Society. 2019;27(4):502-6. Epub 2019/05/08. doi: 10.1016/j.jsps.2019.01.013. PubMed PMID: 31061618; PubMed Central PMCID: PMCPMC6488812.
20. Corey KM, Helmkamp J, Simons M, Curtis L, Marsolo K, Balu S, et al. Assessing Quality of Surgical Real-World Data from an Automated Electronic Health Record Pipeline. Journal of the American College of Surgeons. 2020;230(3):295-305.e12. Epub 2020/01/17. doi: 10.1016/j.jamcollsurg.2019.12.005. PubMed PMID: 31945461.

21. Odeny BM, Njoroge A, Gloyd S, Hughes JP, Wagenaar BH, Odhiambo J, et al. Development of novel composite data quality scores to evaluate facility-level data quality in electronic data in Kenya: a nationwide retrospective cohort study. BMC Health Services Research. 2023;23(1):1139. doi: 10.1186/s12913-023-10133-2.
22. Lasim OU, Ansah EW, Apaak D. Maternal and child health data quality in health care facilities at the Cape Coast Metropolis, Ghana. BMC Health Services Research. 2022;22(1):1102. doi: 10.1186/s12913-022-08449-6.
23. Rumisha SF, Lyimo EP, Mremi IR, Tungu PK, Mwingira VS, Mbata D, et al. Data quality of the routine health management information system at the primary healthcare facility and district levels in Tanzania. BMC Medical Informatics and Decision Making. 2020;20(1):340. doi: 10.1186/s12911-020-01366-w.
24. Gianfrancesco MA, Goldstein ND. A narrative review on the validity of electronic health record-based research in epidemiology. BMC Medical Research Methodology. 2021;21(1):234. doi: 10.1186/s12874-021-01416-5.
25. Byrne E, Sæbø JI. Routine use of DHIS2 data: a scoping review. BMC Health Services Research. 2022;22(1):1234. doi: 10.1186/s12913-022-08598-8.
26. Filippi V, Dennis M, Calvert C, Tunçalp Ö, Ganatra B, Kim CR, et al. Abortion metrics: a scoping review of abortion measures and indicators. BMJ Glob Health. 2021;6(1). Epub 2021/01/31. doi: 10.1136/bmjgh-2020-003813. PubMed PMID: 33514592; PubMed Central PMCID: PMCPMC7849886.
27. Williams CR, Vázquez P, Nigri C, Adanu RM, Bandoh DAB, Berrueta M, et al. Improving measures of access to legal abortion: A validation study triangulating multiple data sources to assess a global indicator. PloS one. 2023;18(1):e0280411. doi: 10.1371/journal.pone.0280411.
28. Stillman M, Kibira SPS, Shiferaw S, Makumbi F, Seme A, Sully EA, et al. Postabortion and safe abortion care coverage, capacity, and caseloads during the global gag rule policy period in Ethiopia and Uganda. BMC health services research. 2023;23(1):104. doi: 10.1186/s12913-022-09017-8.
29. Feng S, Hategeka C, Grépin KA. Addressing missing values in routine health information system data: an evaluation of imputation methods using data from the Democratic Republic of the Congo during the COVID-19 pandemic. Population health metrics. 2021;19(1):44. doi: 10.1186/s12963-021-00274-z.
30. Bernardi FA, Alves D, Crepaldi NY, Yamada DB, Lima VC, Lopes Rijo RPC. Data Quality in health research: a systematic literature review. medRxiv; 2022.
31. Robertson J, Bleiberg J. Hack That Crippled UK Hospitals Highlights Growing Threat to NHS. 2024.
