# Supplementary material for: Assessment of the Quality of post-abortion care records in 20 Public Health Facilities in Uganda: What are the gaps and how can we improve quality?
Source: PLoS One. 2025 Sep 17;20(9):e0329842. doi: 10.1371/journal.pone.0329842 (PMC12443310; doi:10.1371/journal.pone.0329842)
Supplement: Attachment — Submitted filename: Response to Reviewers.docx [file pone.0329842.s004.docx]

**Response to reviewers’ comments**

**Comment 1:** A rebuttal letter that responds to each point raised by the academic editor and reviewer(s). You should upload this letter as a separate file labeled 'Response to Reviewers'.

***Response:*** *Dear editorial team, I have renamed and attached the required files for this resubmission.*

**Comment 2:** A marked-up copy of your manuscript that highlights changes made to the original version. You should upload this as a separate file labeled 'Revised Manuscript with Track Changes'.

***Response: -*** *A marked-up copy file named “Revised Manuscript with Track Changes’ manuscript is attached and uploaded*

**Comment 3:** An unmarked version of your revised paper without tracked changes. You should upload this as a separate file labeled 'Manuscript'.

***Response:*** *Revised unmarked manuscript without track changes named ‘Manuscript’ is attached.*

**Comment 4:** Thank you for stating the following financial disclosure: Funding for this work was secured from Vital Strategies Inc. New York USA. However, the study ended a year ago.

At this time, please address the following queries:

a) Please clarify the sources of funding (financial or material support) for your study. List the grants or organizations that supported your study, including funding received from your institution.

*Response:* *The study was supported by an ‘anonymous donor’ through Vital Strategies Inc., New York, USA.*

No specific funding or material support was received from the authors’ academic or institutional affiliations. The study did not receive any grants or internal funds from Makerere University or the Ministry of Health, Uganda.

b) State what role the funders took in the study. If the funders had no role in your study, please state: “The funders had no role in study design, data collection and analysis, decision to publish, or preparation of the manuscript.”

***Response:*** *Thanks for the advice. We have now stated in the manuscript that ‘The funders had no role in study design, data collection and analysis, decision to publish, or preparation of the manuscript’.*

c) If any authors received a salary from any of your funders, please state which authors and which funders.

***Response****:* *Authors Lynn Atuyambe, Justine Bukenya, Samuel Etajak, Onikepe Owolabi, Sharon Kim-Gibbons, Kristy Friesen, and Arthur Bagonza were partially funded by an anonymous donor.”*

d) If you did not receive any funding for this study, please state: “The authors received no specific funding for this work.”. Please include your amended statements within your cover letter; we will change the online submission form on your behalf.

***Response:*** *Authors* Jesca Nsungwa Sabiiti, Richard Mugahi, and Paul Mbaka received no specific funding for this work

**Comment 5:** When completing the data availability statement of the submission form, you indicated that you will make your data available on acceptance. We strongly recommend all authors decide on a data sharing plan before acceptance, as the process can be lengthy and hold up publication timelines. Please note that, though access restrictions are acceptable now, your entire data will need to be made freely accessible if your manuscript is accepted for publication. This policy applies to all data except where public deposition would breach compliance with the protocol approved by your research ethics board. If you are unable to adhere to our open data policy, please kindly revise your statement to explain your reasoning and we will seek the editor's input on an exemption. Please be assured that, once you have provided your new statement, the assessment of your exemption will not hold up the peer review process

***Response:*** *De-identified data at individual and facility levels will be made available at any time it is requested. However, it is important to note that in Uganda, abortion is outlawed. While post abortion care is acceptable, ability to correctly identify the two concepts (abortion and post abortion care) is hard for many law enforcement officers and policymakers in Uganda. Thus, the data underlying this study contains sensitive health facility-level information, such as the number of PAC cases per health facility. This is why we prefer that relative restriction of the data as advised by the Research Ethics Committee at Makerere University School of Public Health and the Uganda National Council for Science and Technology.*

*Data access requests may be submitted to the School of Public Health Research Ethics Committee via the following email address:* [*sphrecadmin@musph.ac.ug*](mailto:sphrecadmin@musph.ac.ug) *or the corresponding author, and will be considered on a case-by-case basis for researchers who meet criteria for access to confidential data.*

**Comment 6:** Your ethics statement should only appear in the Methods section of your manuscript. If your ethics statement is written in any section besides the Methods, please move it to the Methods section and delete it from any other section. Please ensure that your ethics statement is included in your manuscript, as the ethics statement entered into the online submission form will not be published alongside your manuscript

***Response*** *Thank you for the guidance. We have moved the ethics statement exclusively to the methods section of the manuscript and deleted it from all other sections. The revised manuscript now complies with PLOS ONE’s formatting requirements regarding ethics reporting.*

**Comment 7:** If you are unable to obtain permission from the original copyright holder to publish these figures under the CC BY 4.0 license or if the copyright holder’s requirements are incompatible with the CC BY 4.0 license, please either i) remove the figure or ii) supply a replacement figure that complies with the CC BY 4.0 license. Please check copyright information on all replacement figures and update the figure caption with source information. If applicable, please specify in the figure caption text when a figure is similar but not identical to the original image and is therefore for illustrative purposes only.

***Response****: Thank you for the clarification regarding figure licensing. It will take us a long time to obtain permission to publish the figure under the CC by 4.0 license. As such, given the importance of the data, we have removed the figure from the revised manuscript and updated the in-text references accordingly.*

**Response to Reviewer 1 comments**

**Comment 1:** Page 3, line 80-82: Please add any global or regional evidence to support this statement

***Response:*** *Thank you for the comment. We have revised the sentence on page 3, line 80-82 to include relevant global and regional evidence supporting the statement. The updated text now cites recent studies and global reports highlighting the challenges of data quality in maternal health information systems, particularly in sub-Saharan Africa.*

**Comment 2:** Page 3, Line 97-100 quite same as line 93. I would suggest to modify it to avoid the repetition. Moreover, you can add other kinds of barriers that influence data collection and reporting.

***Response:*** *Thank you for this helpful observation. We have revised lines 97-100 to remove the repetition with line 93 and incorporated additional barriers that influence data collection and reporting. This revised text now provides a broader perspective on the structural and contextual challenges affecting routine data systems, particularly at lower-level health facilities.*

**Comment 3:** Page 4, line 103 Any reference?

***Response****: Thank you for pointing this out. We have now added an appropriate reference to support the statement.*

**Comment 4:** Page 4, line 111 to 112: If conducting this study is your ultimate goal, you should explain how and why the effective data from DHIS2 is crucial for decision-making and budgeting.

***Response:*** *Thank you for the comment. We have revised the sentence on page 4, lines 111-112 to clarify how and why effective data from DHIS2 is critical for decision-making and budgeting. The revised text emphasizes the role of high-quality routine data in informing resource allocation and maternal health program planning at national and district levels.*

**Comment 5:** Page 4, line 116-117: Check this sentence for grammatical error.

*Response: Thank you for highlighting this. We have reviewed and corrected the grammatical error in the sentence on page 4 lines 116-117 to improve clarity and readability.*

**Comment 6:** Page 21, line 357: Please check Figure 3a's description

***Response:*** *Thank you for the observation. We have reviewed and revised the description of figure 3a on page 21, line 357 to ensure it accurately reflects the context of the figure and aligns with the data presented.*

**Comment 7:** Page 21, line 357: Please check the Figure 3a's description

***Response****: Thank you for the observation. We have reviewed and revised the description of figure 3a on page 21, line 357 to ensure it accurately reflects the context of the figure and aligns with the data presented.*

**Comment 8:** Page 27, line 475: Please check the number of countries on DHIS2's platform.

***Response:*** *Thank you for pointing this out. We have verified and updated the number of countries currently using the DHIS2 platform based on the most recent data from the official website (*[*www.dhis2.org*](http://www.dhis2.org)*). The revised sentence reflects the accurate and up-to-date figure, and it now reads “*Currently, over 80 countries around the world use the DHIS2 platform to manage national health information systems, including in many low- and middle-income settings*”.*

**Comment 9:** Page 27, line 483 to 484: How? What can be done to improve DHIS2 and overcome the challenges?

***Response:*** *Thank you for this inquiry. We have revised the sentence on page 27, lines 483-484 to explain how DHIS2 can be improved and what specific actions can help overcome the identified challenges.*

**Comment 10:** Page 29, line 529 (references): Please ensure that the writers keep the same font throughout the whole article.

*Response: Thank you for pointing this out. We have reviewed the manuscript and ensured that the font is consistent throughout, including the reference section.*

**Response to Reviewer 2 comments**

**Comment 1:** PAFP should be written in full.

***Response:*** *Thank you for pointing this out. We have revised the manuscript to spell out PAFP in full upon its first mention as “post-abortion family planning (PAFP)” and have retained the abbreviation for subsequent use throughout the text for clarity and consistency*

**Comment 2:** Authors should note that this result (r =0.083, p<0.001) indicate a very weak relationship albeit positive, thus the evidence is insufficient to submit that there is internal consistency between the records of women who received PAC and those who received PAFP.

***Response:*** *Again, thank you for this observation. We agree that while the correlation between the number of women who received post-abortion care (PAC) and those who received post-abortion family planning (PAFP) was statistically significant (r = 0.083, p<0.01), the strength of the relationship is very weak. We have revised the interpretation of this result in the manuscript to reflect that the statistical significance does not imply strong internal consistence between PAC and PAFP records. The text now more cautiously notes the limited strength of association and emphasizes the need for further investigation into data linkage and recording practices. In part, it reads,* *“Although a statistically significant positive correlation was observed between the number of women who received PAC and those who received post-abortion family planning (PAFP) (r =0.083, p<0.001), the association was very weak suggesting limited internal consistency between the two indicators...”*

**Comment 3: Methods-**Lines 226-230, it is unclear the exact data quality metric the authors meant here as “WHO’s recommended accuracy rate” given that in their Table 2 of data quality metrics, two specifically termed “Accuracy I and II” were listed and none of them have a WHO reference cut-off of 75% (besides completeness of reporting). Please clarify and harmonize.

***Response:*** *Thank you for this important clarification. We acknowledge the ambiguity and have revised lines 226-230 to accurately reflect the appropriate WHO-recommended benchmark. Specifically, the 75% threshold refers to the completeness of reporting as per the WHO Data Quality Review (DQR) toolkit, and not to the metrics labelled as Accuracy I and II in table 2.*

*We have removed the reference to a “WHO-recommended accuracy rate” in this section to avoid confusion and we have harmonized the terminology across the text and Table 2. The accuracy indicators are now clearly described as derived from internal comparisons within the data set, without attributing them to a WHO benchmark.*

**Comment 4:** The description in lines 231-237 appears to indicate that the study was eventually not conducted in 80 facilities, but in 20. If so, the authors should make it clearer and consider the need to redirect the study title, the description of sample determination, and the information in Table 2 such that all pertains to 20 facilities, not 80. This would ensure that readers are not confused or misled. It is suggested that a flow diagram be presented, and the mentioned level of 'accuracy' that informed the final choice and number of facilities that study results are based, be posed as eligibility criteria. If otherwise, then the report in lines 231-237 of the initial assessment of accuracy for all 80 facilities qualify to be considered as part of the study findings and thus be presented in the Results section rather than as a methodological process.

***Response:*** *Thank you and we appreciate your helpful comment. We confirm that although 80 health facilities were initially sampled and reviewed for completeness and consistency, only 20 facilities met the eligibility criteria (≥ 50% discrepancy or missing data) and were therefore included in the final analysis. These 20 were selected for an in-depth audit based on data quality concerns identified during the initial desk review. We have revised the title, abstract, sample, sample size description, table 2 caption, and methods section to clearly state that the final analysis was based on these 20 eligible facilities. We have also added a flow diagram to show the selection process and clarified that the study’s findings are limited to these 20 facilities and not representative of the full sample.*

**Comment 5: Ethical approval -** Approval for two additional districts was mentioned, however the explanation of their eligibility for selection was not provided in the earlier section where the eight districts that met the high and low caseload categories, were listed.

***Response:*** *Thank you for this important observation. We would like to clarify that the two additional areas—Gulu city and Mbarara city were originally part of Gulu district and Mbarara district, respectively. However, during the period of this study, both cities had been granted anv independent city status, thus were no longer administratively under their former districts (Gulu and Mbarara). As a result, it became necessary to obtain separate ethical clearance for these two city jurisdictions, even though their health facilities were initially considered under the original eight-district sampling frame.*

*We have revised the methods section to clearly explain this administrative change and the resulting need for additional ethical approvals.*

**Comment 6: Results -** As mentioned, it needs to be established what number of facilities the results are based on. That said, the convention in health studies is to report both absolute numbers (frequency) along with percentages (proportion); authors should reflect both.

***Response:*** *Thank you for this comment. Our sample consisted of 80 health facilities randomly chosen across four regions to ensure geographical diversity and stratified by caseload to capture contrasting report contexts (high vs low PAC volumes). The selection of 20 facilities in stage 2 for in-depth validation was guided by predefined criteria of data quality concern (i.e. facilities with ≥ 50% discrepancies or missing data).*

**Comment 7:** For the comparison between districts or facility type, the report would be better served by computing statistical tests of significance to allow for statistical inference and generalization of findings, given that only a subset of facilities was selected for each stratum (district or facility type).

***Response:*** *Thank you for this comment. We acknowledge the importance of statistical testing in enabling generalization. However, in this study, statistical tests of significance were not applied when comparing districts or facility types because the primary goal was not to make population-level inferences, but rather to assess data quality patterns using purposefully stratified random samples for exploratory validation. Our methodological approach aligns with WHO’s Data Quality Review (DQR) toolkit, which recommends stratified assessment of data quality indicators not necessarily requiring inferential statistics when the objective is diagnostic rather than representative. Therefore, while statistical significance testing may be appropriate in population-representative studies, our study aimed at identifying data quality challenges in typical operational settings to inform system-level improvements, not to generalize findings across all facilities or districts.*

**Comment 8:** In Figure 1 and in the rest of the narrative, what ‘average’ was computed as percentages? This should be stated beforehand under a Data Analysis section, which appears to be missing. Authors should address this by providing that subsection under Methods.

***Response:*** *Dear reviewer, Figure 1 is a map of Uganda, which we have removed because we were unable to obtain the base files used to create this map. he term “Data quality assessment” instead of “Data analysis”. Table 2 is a summary of how the indicators were analysed.*

**Comment 9:** Relating to this, a computation of regression coefficient for internal consistency was stated in the Abstract, again there is no details offered in a Data Analysis section.

***Response:*** *Thank you, our dear reviewer. We have included, under the data quality assessment section, how internal consistency was computed*

***Comment 10:* Completeness of reporting and timeliness -** The description of what was assessed as completeness is not clear and seems different from the WHO criterion. In Lines 279-282, it appears that authors evaluated data completeness by reviewing whether DHIS data were correct with what was sent to the district health information officer. If so, it does not align with the WHO criterion on Table 1, thus should be re-evaluated and reported correctly.

***Response****: Thank you for pointing out this oversight, our dear reviewer. We now describe the completeness of reporting as follows: We evaluated the completeness of facility reporting by calculating the total number of health facilities expected to submit monthly reports with PAC data (denominator) versus the actual number of health facilities submitting reports with PAC data (numerator). According to WHO cut-off of 75%, if a district had less than 75% of the facilities not reporting, on average, that district was low reporting as shown in table 1.*

**Comment 11**: There is another metric for assessing consistency between data sources that appears in authors’ Table 1 as “Accuracy II” – in which case the primary data source is the facility records (not the district officer’s). There is a subsection on Accuracy between original facility data and DHIS2 data (Accuracy II) – see comments below on Figures 3a – 3c.

Authors could consider a separation of the results for completeness and timeliness though it is understandable that the latter has very little information to report.

***Response****: We appreciate this comment, our dear reviewer. We have explained how we determined the accuracy that is reported in Figures 3a-3c as follows: To assess the accuracy of reported data, we compared values for selected indicators documented in the original facility registers with corresponding values reported in the DHIS2 system multiplied by 100. An accuracy score of 100% indicated perfect agreement between the two sources. Scores greater than 100% indicated underreporting of health facility data in the DHIS2 system, while scores less than 100% indicated overreporting of health facility data in the DHIS2 system.*

*In addition, we have separated the results for completeness and timeliness as advised.*

**Comment 12:** **Consistency over time and Table 3 -** Lines 310-312: authors should change the term “match” at it communicates a different meaning from what a consistency check attempts to establish. As much as possible, deductive statements should be reserved for the Discussion section.

***Response:*** *Thank you for this comment. We agree that the term “match” may imply exact agreement, which is not the intended interpretation of a consistency check, especially when using WHO-recommended ratio thresholds. We also appreciate the suggestion to reserve deductive language for the discussion section. In response, we have revised the sentence to more accurately reflect the nature of the consistency check. The term “match” has been replaced with more neutral language, and the interpretation has been framed descriptively rather than deductively.*

**Comment 13: Internal consistency of reported data by checking for outliers in DHIS2 Data**
This subheading should be changed. This metric was labeled “Outliers in reference year” in the methods section, authors should harmonize use of terminologies and reconcile methods used to evaluate any of the metrics with what had been earlier stated as WHO criteria. Thus, authors should be clear on whether they used the module on DHIS-2 platform that performs outlier detection and generates the frequency of occurrence. Otherwise, they should describe any other outlier analysis method that was used.

***Response****: Thank you for this constructive feedback. We agree that consistency in terminology is essential for clarity and methodological rigor. In response, we have updated the subheading from “Internal consistency of reported data by checking for outliers in DHIS2 Data” to “Outliers in reference year” to align with the terminology used in the methods section and the WHO data quality review (DQR) toolkit.*

**Comment 14:** Again, only aggregates (totals per year, per indicator) at district levels were analyzed (Tables 4 & 5). Authors should prioritize an analysis for all sampled facilities, as the first instance. This demonstrate fidelity to the thrust of the paper, which is a report of data quality performance at facility level.

***Response:*** *Thank you very much, our dear reviewer. We have prioritized the analysis of sampled health facilities as the first instance and then districts next to demonstrate the fidelity to the thrust of the paper. Table 4 is now table 5 and table 5 is now table 4.*

**Comment 15: Consistency of reported data in DHIS2**
This subheading should be changed. Again, there is a need to be consistent and harmonize use of terms: this metric was labeled “Accuracy I” which was described as “Consistency between related data the extent to which the values for two or more PAC indicators exhibit the predicted relationship”. Additionally, the WHO method to evaluate it is different from what was shown in the analysis (Table 6). If the WHO method of evaluating and rating any metric was modified, this should be pre-informed in the Methods section.

***Response****: We very much appreciate this constructive comment. We agree that the terminology used in the subheading should be consistent with the definitions presented in Table 2 and throughout the manuscript. Accordingly, we have revised the subheading “consistency of reported data in DHIS2” to “Accuracy I: consistency between related PAC indicators” to align with our earlier definitions and WHO’s categorization of internal consistency metrics. Regarding the methods used to assess this metric, we acknowledge that while the WHO data quality review toolkit recommends using ratios between related indicators (with a tolerance of ±10) to assess internal consistency, our analysis extended this by incorporating Pearson correlation coefficients to evaluate both the strength and direction of the relationship over time. This approach was adopted to enhance interpretability in our specific context, where historical data on some PAC indicators were missing or sparse in earlier years*

**Comment 16:** The authors’ use of Pearson’s correlation analysis however had some errors. First, Table 6 title suggested it as ‘predicting’ a relationship between the indicators, which is not what this evaluation is about, and besides correlation is not for ‘prediction’ analysis (as against regression). Second, the correlation coefficients were wrongly interpreted as “strong correlation” whereas none fell between 0.5 and 0.9 regardless of having significant P-values.

***Response:*** *Thank you for pointing this out. We appreciate the clarification regarding the appropriate use and interpretation of Pearson’s correlation coefficients. In response:*

1. *Correction of table title: We acknowledge that the original title of Table 6 may have implied a predictive relationship. To avoid confusion, we have revised the table title to: “Table 6: Annual ratios and correlation between post-abortion family planning and antenatal care indicators”. This now correctly reflects that the analysis examined associations rather than prediction.*
2. *We also agree that the wording used to describe the strength of the correlations was imprecise. None of the coefficients reported in Table 6 meet the conventional threshold for moderate (0.3-0.5) or strong (0.5-0.9) correlation. We have revised the results and discussion sections to describe these as weak but statistically significant positive correlations and removed any reference to them being strong.*

**Comment 17:** **Accuracy between original facility data and DHIS2 data**
This metric was properly labeled but should further be reflected as “Accuracy II” to harmonize the thought process from Methods to Results. In addition, authors should reconcile the method used (appears to be Trend Consistency Analysis) versus what was described as a WHO criterion (Consistency Ratios) in the Methods section. As earlier mentioned, any modification in approach should have been described in the Methods.

***Response:*** *Thank you for this valuable comment. We acknowledge the need for terminological and methodological consistency between the methods and results sections. In response:*

*1. We have revised the results subheading to read “Accuracy II: Consistency between facility register data and DHIS2 reports”. This aligns with the terminology used in Table 2 and the methods section to ensure clarity and consistency throughout the manuscript.*

*2. We appreciate the reviewer’s observation that the figures (3a-3c) may resemble trend consistency analysis. However, as described earlier, our analysis followed the WHO DQR definition of Accuracy II—i.e. comparing values from original facility registers to those reported in DHIS2, expressed as a percentage. The plotted trends were used for visual representation of discrepancies over time, not for trend consistency analysis perse.*

**Comment 18:** Authors stated that records from facilities were presented as percentages of accuracy, but it is not clear how they was actually derived. This should be explained.

***Response:*** *Thank you for pointing this out. We have rectified this as follows: To assess the accuracy of reported data, we compared values for selected indicators documented in the original facility registers with corresponding values reported in the DHIS2 system multiplied by 100. An accuracy score of 100% indicated perfect agreement between the two sources. Scores greater than 100% indicated underreporting of health facility data in the DHIS2 system, while scores less than 100% indicated overreporting of health facility data in the DHIS2 system.*

**Comment 19***:* That said, the methodology used, which appears like Trend Consistency Analysis, is ideally for testing Accuracy I (consistency between separate but related indicators from one data source) i.e. the previous metric/subheading. This is really what is shown in Figure 3a or 3b where two separate, but related indicators were plotted side-by-side on a line graph. However, the authors reached too far by comparing the pattern from two separate graphs as basis to analyze data accuracy between two data sources - facility data and DHIS data. Hence, authors should interpret Figures 3a-c as evaluation of Accuracy I using Trend Consistency Analysis and move that under the previous section. The implication would be that three methods were used to evaluate consistency between related indicators: correlation analysis of means of data indicators; trend consistency analysis, and WHO Ratios (which weren’t computed).

***Response:*** *We appreciate the reviewer’s observation that the figures (3a-3c) may resemble trend consistency analysis. However, as described earlier, our analysis followed the WHO DQR definition of Accuracy II—i.e. comparing values from original facility registers to those reported in DHIS2, expressed as a percentage. The plotted trends were used for visual representation of discrepancies over time, not for trend consistency analysis perse.*

**Comment 20:** The information in Table 7 is not clear; are they proportions or ratios or averages, and how were they calculated?

***Response****: Dear reviewer, we clarify that to assess the accuracy of reported data, we compared values for selected indicators documented in the original facility registers with corresponding values reported in the DHIS2. The information presented is ratios.*

**Comment 21: Consistency between household survey and DHIS2 data**
This entire section could be deleted altogether as there was no information, and more importantly, it was not mentioned as part of the evaluation dimensions.

***Response:*** *Thank you once again for pointing this out. We have deleted this section altogether since there was no information and was not mentioned as part of the evaluation dimensions.*

**Comment 22: Discussion**
This section should be revised in line with any modifications made to the Results section.

***Response:*** *We have revised the discussion section in line with the modifications made to the results.*

********************************************************************************

**---End---**
